# Focal vibrations enhance somatosensory facilitation in healthy subjects: A pilot study on Equistasi^®^ and high-frequency oscillations

**DOI:** 10.3389/fneur.2022.1052989

**Published:** 2022-11-23

**Authors:** Alessandro Cruciani, Jacopo Lanzone, Gabriella Musumeci, Vincenzo Di Lazzaro, Massimo Marano

**Affiliations:** ^1^Research Unit of Neurology, Neurophysiology and Neurobiology, Department of Medicine, Università Campus Bio-Medico di Roma, Rome, Italy; ^2^Operative Research Unit of Neurology, Fondazione Policlinico Universitario Campus Bio-Medico, Rome, Italy; ^3^Istituti Clinici Scientifici Maugeri IRCCS, Neurorehabilitation Unit of Milan Institute, Milan, Italy

**Keywords:** focal vibration, high-frequency oscillation, movement disorders, non-pharmacological rehabilitation, Equistasi^®^, somatosensory processing

## Abstract

**Background:**

Equistasi^®^ is a vibrotactile device composed of nanotechnology fibers that converts temperature change into mechanical energy by self-producing a focal vibration. It is used in non-pharmacological rehabilitation in patients with movement disorders and multiple sclerosis sequelae. Nonetheless, the mechanism underlying such an improvement in motor functions is still poorly understood.

**Objectives:**

We designed a small uncontrolled pilot trial to explore the effect of Equistasi^®^ on the somatosensory pathway through the analysis of high-frequency oscillations (HFOs).

**Methods:**

For all the included subjects, we recorded somatosensory-evoked potentials (SEPs) at the baseline (T0) and at 60 min after the application of Equistasi^®^ (T1) on the seventh cervical vertebra level and at the forearm over each flexor carpi radialis, bilaterally. Then, we extracted the HFOs from the N20 signal and compared the HFO duration and area under the curve pre- and post-Equistasi^®^ application.

**Results:**

In a head-to-head comparison of T0 to T1 data, there was a statistically significant reduction in the total HFO area (*p* < 0.01), which was prominent for the late component (*p* = 0.025). No statistical differences have been found between T0 and T1 HFO duration (*p* > 0.05). We further evaluated the N20 amplitude from the onset to the N20 peak to avoid possible interpretational bias. No statistical differences have been found between T0 and T1 (*p* = 0.437).

**Conclusion:**

Our clinical hypothesis, supported by preliminary data, is that vibrotactile afference delivered by the device could work by interfering with the somatosensory processing, rather than by peripheral effects.

## Introduction

Equistasi^®^ is a vibrotactile device composed of nanotechnology fibers. When this small tool is worn on the body, it converts the temperature change, due to the contact with the skin, into mechanical energy by self-producing a focal vibration (1). Applying an Equistasi^®^ device over a muscle tendon supposedly modulates the Golgi mechanoreceptor activity, which is recognized as the proprioceptive system gate. The application of Equistasi^®^ in clinical practice is supported by randomized trials conducted on movement disorders, such as Parkinson's disease or multiple sclerosis sequelae (1, 2). Its effect is mainly observed in balance, gait, and overall motor function and is likely mediated by sensory feedback modulation of the proprioceptive system. Nevertheless, the mechanism underlying such an improvement in motor functions is still poorly understood. Somatosensory pathways and sensory motor integration have a pivotal role in modulating the motor output, as also suggested by the application of sensory cueing in movement disorders, such as Parkinson's disease or dystonia (i.e., sensory tricks) (3). Nevertheless, the complete mechanisms of action are still not fully understood, and studies that aim at investigating the neurophysiological basis of proprioceptive devices on somatosensory pathways are lacking. High-frequency oscillations (HFOs) are a well-established neurophysiological marker to evaluate somatosensory processing (4, 5). HFOs are fast physiological oscillations that underpin somatosensory-evoked potential. These waves are obtained by applying digital high-pass filtering on low-frequency median SEP to divide the signal from the original N20 response (6). Such oscillations are subdivided into an early and a late component based on the peak of the N20. Early HFOs measure thalamocortical input, while late HFOs reflect the activity of intracortical GABAergic interneurons located in the somatosensory cortex (6). Hence, we designed a small uncontrolled pilot trial to explore the effect of Equistasi^®^ on somatosensory processing through the evaluation of high-frequency oscillations (HFOs), which change pre- and post-Equistasi^®^ applications.

## Methods

### Participants

A total of 10 right-handed healthy volunteers (four women, six men; median age 21.5 ± 2.9 years), were consecutively enrolled by the school of medicine of our university. The handedness of the participants was tested by using the Edinburgh Handedness Inventory (7). The study was performed in accordance with the Declaration of Helsinki and was approved by the local ethics committee. The participants signed a regular informed consent.

### Study design

For all the enrolled subjects, we recorded somatosensory-evoked potentials (SEPs) at the baseline (T0) and at 60 min after the application of Equistasi^®^ (T1).

The device was applied over the skin at the 7th cervical vertebra level as suggested by the manufacturer, and at the forearm over each flexor carpi radialis (i.e., a median nerve innervated muscle), bilaterally.

### Neurophysiological assessment

The median nerve SEP was evoked by conventional electrical stimulation at the wrist of the dominant hand using a high-voltage stimulator (DS7A, Digitimer Ltd, UK). We used bar electrodes; the anode was placed on the wrist crease, while the cathode was placed proximally. In total, 1,200 pulses of 200-μs duration were delivered at a frequency of 1.9 Hz. We used the lower intensity capable of generating a slight thumb twitch. Ag/AgCl surface electrodes were placed at left CP3 (active electrode) and Fz (reference electrode) locations of the international 10/20 system. The 1,200 sweeps were averaged, bandpass-filtered (0.5–2,000 Hz), and digitized at a sample rate of 5 kHz using a portable amplifier (BrainVision Recorder, BrainAmp MR plus, Brain Products GmBH, Germany, version:1.10).

### Data analysis

Data were analyzed by *ad hoc* MATLAB (MathWorks, Inc., Massachusetts, USA, version: R2021b) script. A digital 400–800 Hz bandpass Butterworth filter was applied to extract HFOs. The area and duration were calculated from the rectified data from the point at which upward deflection was more than 50% of the background noise to the point where deflection was < 50% (8). The N20 features and the HFO area and duration were then compared across conditions.

### Statistical analysis

The statistical analysis was performed using SPSS (IBM Corp., Armonk, New York, USA, version 25). All the results are expressed as mean ± standard deviation. Data were compared using the Wilcoxon signed-rank test or the *t*-test for paired data according to their distribution (Shapiro–Wilk test) and were corrected for multiple comparisons according to the Benjamini–Hochberg procedure, with a false discovery rate of 0.05. The experiment was well tolerated, and no dropouts were reported.

## Results

**Figure 1:**
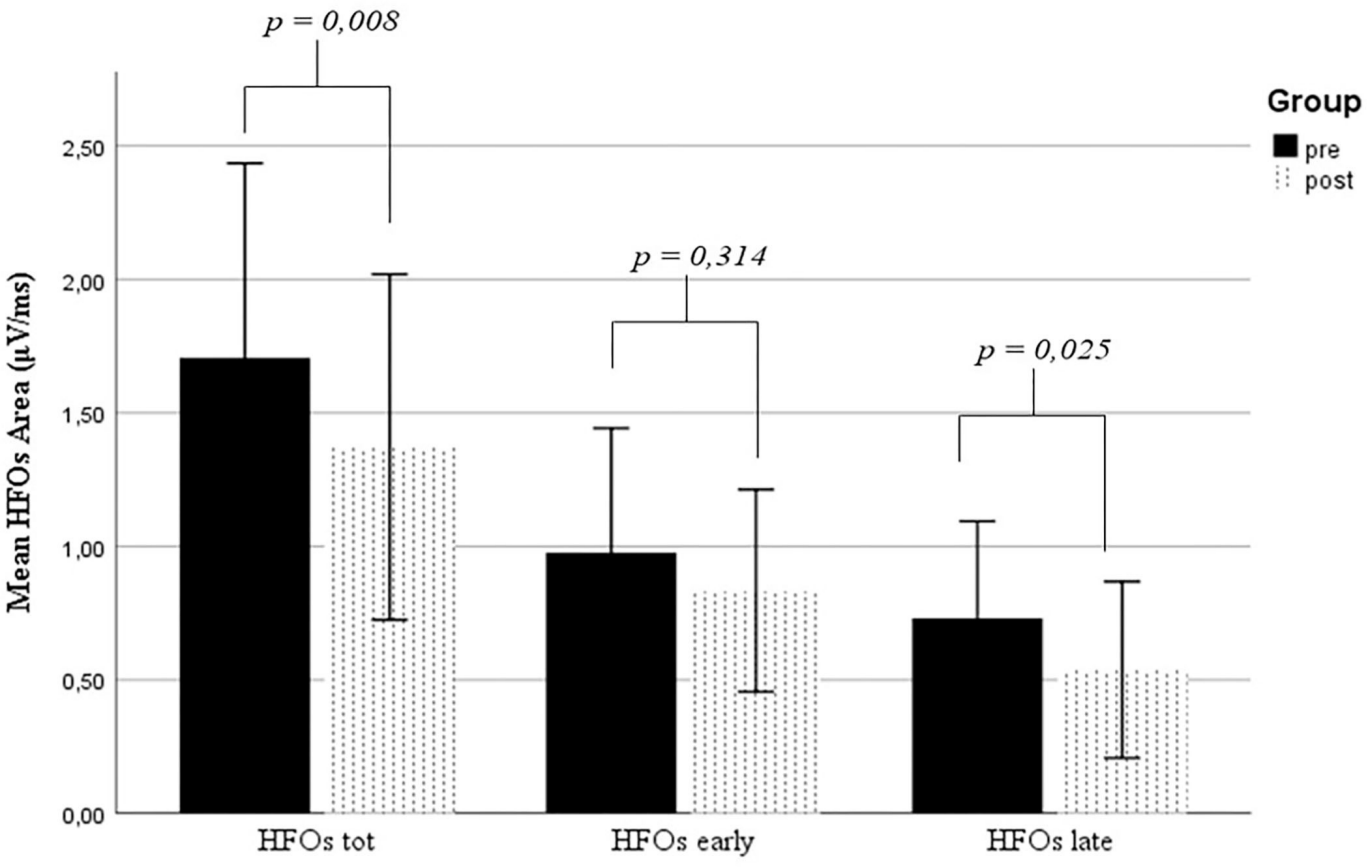
Comparison of HFO values between T0 and T1. HFOs, high-frequency oscillations.

The N20 latency was 19.6 ms ± 1.6 at the baseline (T0) and remained stable at T1. At the baseline (T0), the HFO presented a total AUC of 1.705 μV/ms (SD = ±1.153), subdivided into early (0.975 μV/ms ± 0.737), and late (0.730 μV/ms ± 0.574) HFOs. Similar findings were observed at T1 (total AUC = 1.372 μV/ms ± 1.023; early AUC = 0.834 μV/ms ± 0.598; late AUC = 0.537 μV/ms ± 0.522). In a head-to-head comparison of T0 to T1 data, there was a statistically significant global reduction in the total HFO area (*p* < 0.01), which was prominent for the late component (*p* = 0.025) (Figure 1). No statistical differences have been found between T0 and T1 HFO duration (*p* > 0.05). The latter showed a total value of 8.8 ms ± 1.9 and 8.3 ms ± 2 at T0 and T1, respectively. The early HFO duration at T0 was 4.8 ms ± 1.3 and 4.5 ms ± 1.5 at T1, while the late HFO duration was 2.8 ms ± 2.2 at T0 and 2.5 ms ± 2.1 at T1. We further evaluated the N20 amplitude from the onset to the N20 peak to avoid possible interpretational bias. At T0, the onset-to-peak N20 amplitude was 1.8 μV ± 0.8, while at T1, the amplitude was 1.9 μV ± 0.9. No statistical differences have been found comparing the onset-to-peak N20 amplitude between T0 and T1 (*p* = 0.437). The main results are summarized in Table 1.

**Table 1:** HFOs and N20 values expressed as means ± standard deviation.

	**T0**	**T1**	***P*-value**
Total HFOs AUC (μV/ms)	1.705 ± 1.153	1.372 ± 1.023	0.008
Early HFOs AUC (μV/ms)	0.975 ± 0.737	0.834 ± 0.598	0.314
Late HFOs AUC (μV/ms)	0.730 ± 0.574	0.537 ± 0.522	0.025
Total HFOs duration (ms)	8.8 ± 1.9	8.3 ± 2	0.075
Early HFOs duration (ms)	4.8 ± 1.3	4.5 ± 1.5	0.089
Late HFOs duration (ms)	2.8 ± 2.2	2.5 ± 2.1	0.352
Onset-to-peak N20 Amplitude (μV)	1.8 ± 0.8	1.9 ± 0.9	0.437

HFOs, high-frequency oscillations; AUC, area under the curve.

## Discussion

HFO is a powerful neurophysiological tool to assess the integrity of the somatosensory pathway (5). Since the cortical (i.e., late) part derives from intracortical GABAergic interneurons, and the early part reflects the thalamocortical section, HFO is widely used in the study of pathophysiological mechanisms in which alteration of the somatosensory system is believed to be a pathophysiological mechanism (8–13). One of the first applications of HFO in the clinic practice was in the field of epilepsy (9, 10). In conditions such as juvenile myoclonus epilepsy (JME) or familial adult myoclonic epilepsy type 2 (FAME2), the alteration of HFOs, especially in the cortical parts, help identify the contribution of the somatosensory system hyperexcitability in the epileptic susceptibility of the patients (9, 10). Furthermore, studies conducted on patients with Parkinson's disease (11, 13) and cervical dystonia found a modification in the late HFO component. Specifically, patients with PD showed enhanced HFOs than healthy controls (11, 12). One possible explanation for the enhanced HFOs is based on the interaction between the basal ganglia and the somatosensory system in patients with PD. Indeed, in patients with PD, the GABAergic impairment of neurons located in the external part of the globus pallidus could lead to disinhibition of GABAergic interneurons in the thalamic reticular nucleus. Subsequently, the thalamocortical projection is diminished, causing a reduction in the activity of interneuron layer IV in the sensory cortex (11, 12), which are believed to be the generators of late HFOs (14). Interestingly, contrasting results have been found for patients with dystonia, with a decrease in GABAergic activity leading to smaller HFOs (12). Hence, in patients with dystonia, the device could interact with the aforementioned cortical pathways, restoring the physiological equilibrium. In our study, we found significant effects of Equistasi^®^ application on the cortical part of HFOs (AUC reduction of ~25%), similarly to that observed in rTMS experiments (15). One hypothesis is that the vibrotactile stimulation delivered by Equistasi^®^ generates a sensory signal that enters the central nervous system from the Golgi mechanoreceptors. Thus, the vibrotactile signal passes through the dorsal column and ultimately terminates in the ventral posterolateral (VPL) nucleus of the thalamus. Here, it takes synapses with GABAergic neurons (16).

## Conclusion

Indeed, our clinical hypothesis supported by preliminary data is that vibrotactile afference delivered by the device could work by interfering with somatosensory processing, rather than by peripheral effects. The interpretation of our results is limited by the small population and uncontrolled study design and deserves further experiments. Another limitation is the median age of the population, which is not completely representative of the typical age of patients with neurological diseases, such as Parkinson's disease or dystonia. This difference makes our study findings difficult to be generalized to neurological disorders. However, if proven true, the reduction in the late HFO part induced by Equistasi^®^ might be hypothetically of help in restoring the equilibrium between somatosensory and motor pathways, which have been thoroughly investigated in Parkinson's disease, dystonia, and related disorders. The potential application of this device in patients with dystonia is particularly interesting since there is an acceptability issue in current therapies—that is, botulinum toxin. Indeed, Equistasi^®^ could represent a non-invasive valid option in dystonia treatment. Moreover, our study considers one neurophysiological tool, so future studies should consider other parameters, such as short intracortical inhibition (SICI) and short afferent inhibition (SAI). Hence, future controlled trials with a more conspicuous sample size evaluating the effects of the device in healthy subjects and in patients with dystonia and PD are needed to confirm this preliminary data.

## Data availability statement

The raw data supporting the conclusions of this article will be made available by the authors, without undue reservation.

## Ethics statement

The studies involving human participants were reviewed and approved by Università Campus Bio-Medico di Roma. The patients/participants provided their written informed consent to participate in this study.

## Author contributions

MM: conceptualization. AC, JL, GM, VD, and MM: methodology. VD and MM: supervision. AC and MM: writing—review and editing. All authors contributed to the article and approved the submitted version.

## Funding

This study was supported by Equistasi^®^. The funder was not involved in the study design, collection, analysis, interpretation of data, the writing of this article, and the decision to submit it for publication.

## Conflict of interest

The authors declare that the research was conducted in the absence of any commercial or financial relationships that could be construed as a potential conflict of interest.

## Publisher's note

All claims expressed in this article are solely those of the authors and do not necessarily represent those of their affiliated organizations, or those of the publisher, the editors and the reviewers. Any product that may be evaluated in this article, or claim that may be made by its manufacturer, is not guaranteed or endorsed by the publisher.
